# Stress, coping, resilience, and sleep during the COVID‐19 pandemic: A representative survey study of US adults

**DOI:** 10.1002/brb3.2384

**Published:** 2021-10-17

**Authors:** Andrew T. Gargiulo, Laurel M. Peterson, Laura A. Grafe

**Affiliations:** ^1^ Department of Psychology Bryn Mawr College Bryn Mawr Bryn Mawr Pennsylvania USA

**Keywords:** coping, coronavirus, COVID‐19, gender differences, pandemic, sleep, stress

## Abstract

**Introduction:**

The COVID‐19 pandemic is a global health emergency resulting in widespread death and substantial disruption to daily life. Previous research has shown that novel disease outbreaks are associated with high stress levels and sleep impairments that lead to neuropsychiatric consequences. Therefore, it is vital to study both stress and protective factors such as coping and resilience that may hinder or help sleep quality during the COVID‐19 pandemic. Further, as gender disparities exist in sleep quality, it is important to understand the relationship between pandemic‐related stress, coping strategies, resilience, and sleep in bothgenders during the COVID‐19 pandemic.

**Methods:**

Our study examined how gender, stress, coping, and resilience were associated with sleep cross‐sectionally during the COVID‐19 pandemic in a representative sample of US adults (*N* = 393).

**Results:**

Consistent with many recent studies, we found that worsened sleep quality in women compared to men persisted during the COVID‐19 pandemic. Interestingly, pandemic‐related stress was not significantly associated with sleep quality, but pandemicrelated coping was associated with sleep independent of robust controls and trait resilience.

**Conclusions:**

Greater primary control engagement coping was associated with better sleep quality, while involuntary engagement coping was associated with poor sleep quality. Future research should extend the findings with actigraphy and explore ways to enhance beneficial coping and sleep health during pandemics.

## Abstract 2

Our study examined how gender, stress, coping, and resilience were associated with sleep during the COVID‐19 pandemic in a representative sample of US adults. We found that women experienced worse sleep quality compared to men. Interestingly, pandemic‐related stress was not significantly associated with sleep quality, but pandemic‐related coping associated with sleep independent of robust controls and trait resilience. Specifically, greater primary control engagement was associated with better sleep quality, while involuntary engagement coping was associated with poor sleep quality.

## 1 INTRODUCTION

The novel coronavirus has generated an unprecedented global public health emergency. COVID‐19, the respiratory disease caused by the coronavirus, is highly infectious, and has resulted in widespread death (Dhama et al., 2020; Szcześniak et al., 2021). Thus, there is fear and anxiety in contracting the disease among the general population (Eder et al., 2021; McKay et al., 2020; C. Wang et al., 2020). In addition, government‐mandated quarantining and social‐distancing measures have been used to contain the spread of the virus, which has caused substantial disruption to daily life (Brooks et al., 2020; Chu et al., 2020). The fear of exposure to the virus and downstream societal disruptions due to the pandemic (such as financial instability due to job loss, isolation from friends and family, and changes to normal routines) are significant and pervasive stressors (Chu et al., 2020).

High levels of stress are associated with reduced sleep quality (Dahlgren et al., 2005; Hoge et al., 2011; Tousignant et al., 2019). Likewise, sleep impairments are a common feature of stress‐related psychiatric disorders (Benca, 1996; Breslau et al., 2004; Chang et al., 2019; Ross et al., 1989; Zhang et al., 2020). Previous studies have examined how past infectious disease outbreaks have influenced stress and sleep quality. Specifically, large outbreaks of novel infectious diseases such as Severe Acute Respiratory Syndrome (SARS) and Middle Eastern Respiratory Syndrome (MERS) were associated with high levels of stress and anxiety (Khalid et al., 2016; Leung et al., 2003) as well as sleep disruptions (S. M. Lee et al., 2018; Yu et al., 2005) amongst healthcare workers and the general population.

The recent literature has demonstrated that stress during the COVID‐19 pandemic is associated with impaired sleep. For example, studies conducted on college students during the pandemic revealed that stress predicts greater latency to fall asleep (Benham, 2020; Gas et al., 2021; Saraswathi et al., 2020; X. Wang et al., 2020). In addition, among healthcare workers, a strong relationship between stress and poor sleep quality have been reported (Al Maqbali & Al Khadhuri, 2021; Jahrami et al., 2020; Kim‐Godwin et al., 2021). Importantly, the association between stress and sleep during the pandemic has been reported in diverse populations (Blume et al., 2020; Casagrande et al., 2020; L. Li et al., 2020), but more research is needed to examine whether these findings extend to the general US population. Moreover, previous research studies have not thoroughly addressed relevant covariates, such as regional virus severity and socioeconomic status, to isolate the stress and sleep relationship. Lastly, the majority of research on coronavirus has assessed stress using general stress measures, which precludes conclusions about the influence of the stress of COVID‐19 on sleep. Thus, in order to expand emerging findings on stress and sleep during the coronavirus pandemic, our research study examines COVID‐19‐related stress and sleep among the US population, accounting for socioeconomic status and regionalized virus severity.

Though there is rich literature describing the association between stress and sleep within the COVID‐19 context (Alimoradi et al., 2021), less is known about the role of coping and resilience in determining sleep outcomes during the COVID‐19 pandemic. While coping refers to both cognitive and behavioral strategies to manage stressful events (Folkman & Moskowitz, 2004), resilience refers to the adaptive capacity to bounce back or recover from stressful situations (Smith et al., 2008; Steinhardt & Dolbier, 2008). It is possible that coping and resilience may play a larger role in sleep outcomes than the stress of the COVID‐19 pandemic itself. Consequently, it is crucial to study these variables together, to gain a full picture of how our sleep may be affected during the COVID‐19 pandemic.

Previous research (outside of a pandemic) indicates that coping strategies can directly influence sleep quality (Pillai et al., 2014). Coping strategies can be adaptive or maladaptive (Compas et al., 2017; Conklin et al., 2015; Meng & D'Arcy, 2015). Adaptive strategies such as primary control engagement coping (which focuses on directly altering the stressor or one's response to it, e.g., through problem solving) and secondary control engagement coping (which focuses on adaptation to the problem, e.g., through cognitive restructuring) have been shown to attenuate the risk of adverse mental health outcomes and sleep impairments (Carver et al., 1989; Connor‐Smith et al., 2000; Finnell et al., 2017; Lancee et al., 2010; Morin et al., 2003; Rosenberg et al., 2011; Veenema et al., 2003; Wood et al., 2010). On the other hand, maladaptive strategies such as disengagement coping (viewed as attempts to suppress arousal, e.g., through avoidance), involuntary engagement coping (viewed as involuntary emotional and physiological stress, e.g., through intrusive thoughts and sympathetic arousal), and involuntary disengagement coping (which includes efforts to orient away from one's emotions, e.g., through emotional numbing) have been associated with poor psychiatric outcomes and sleep disturbances (Carney et al., 2006; Compas et al., 1997; Connor‐Smith et al., 2000; Held et al., 2011; Kwan et al., 2014; S. Lee et al., 2019; Matthews et al., 2016; Tobin et al., 1989). Elucidating how coping strategies influence sleep during a global pandemic could be useful from a theoretical as well as an applied perspective. Given that coping skills can be enhanced with training, understanding which specific coping strategies promote the best quality sleep may support physical and mental health during a stressful global pandemic.

Similar to adaptive coping strategies, the literature indicates that a high level of resilience may protect against stress‐induced sleep impairments (G. Li et al., 2016; Liu et al., 2016; Palagini et al., 2018; J. Wang et al., 2020) and stress‐related psychiatric disorders (Shrivastava et al., 2019). Recent research demonstrated that higher psychological resilience weakened the relationship between stress on sleep quality in both college students and healthcare workers (Du et al., 2020; Labrague, 2021). These results suggest that resilience is an important protective factor for sleep during pandemics in these particular populations. However, it remains unclear how resilience is associated with sleep in more diverse populations during the coronavirus pandemic. To address this, the present study will examine how resilience is associated with sleep in a representative sample of the United States.

The emerging literature has explored gender differences in sleep quality during the COVID‐19 pandemic. Similar to non‐pandemic conditions (Baker et al., 2007; Mallampalli & Carter, 2014; Ohayon et al., 2013), the literature indicates that women report worse sleep quality than men during the COVID‐19 pandemic in diverse populations (Cellini et al., 2021; Franceschini et al., 2020; Paiva et al., 2021; Salfi et al., 2020; Siddique et al., 2021). Moreover, women report greater insomnia, including longer latencies to fall asleep and more sleep disturbances, compared to men during the COVID‐19 pandemic (Casagrande et al., 2020; Del Río‐Casanova et al., 2021; Lin et al., 2021). As sleep impairments are an important phenotype in stress‐related disorders (Ross et al., 1989), which are more common in women (Kessler et al., 1994; Nestler et al., 2002), it is important to understand the associations between pandemic‐related stress, coping strategies, resilience, and sleep in both genders during the COVID‐19 pandemic.

Consequently, we sought to explore how stress and protective factors such as adaptive coping and resilience may hinder or help sleep quality during the COVID‐19 pandemic in men and women in the United States. In the present study, we measure pandemic‐related stress in a representative sample of the United States, while accounting for robust demographic and COVID‐19 contextual factors. Specifically, we control for subjective socioeconomic status and unemployment, which is especially salient to stress and sleep quality during a pandemic with extreme financial burden and high rates of job loss (Rudenstine et al., 2021; Nicola et al., 2020). In addition, we control for coronavirus epidemiological infection and mortality rates for the location in which each participant resided at the time of the survey, as regional virus severity could affect the stress and sleep relationship.

Our study collected self‐report assessments of pandemic‐related stress, coping, resilience, and sleep quality in a representative sample of adults living in the United States in June of 2020 during the COVID‐19 pandemic. We anticipated that greater pandemic‐related stress would be associated with poorer global sleep quality, longer latency to fall asleep, and poorer subjective sleep quality. Further, we expected that maladaptive stress responses including involuntary engagement coping would be associated with poorer global sleep quality, longer sleep latency, and poorer subjective sleep quality. Conversely, we hypothesized that higher resilience and greater use of adaptive coping strategies such as primary control engagement coping would be associated with better global sleep quality, shorter sleep latency, and better subjective sleep quality. Lastly, given the recent literature demonstrating gender disparities in sleep quality during the COVID‐19 pandemic (Casagrande et al., 2020; Cellini et al., 2021; Del Río‐Casanova et al., 2021; Franceschini et al., 2020; Lin et al., 2021; Paiva et al., 2021; Salfi et al., 2020; Siddique et al., 2021), we hypothesized that women would experience lower global sleep quality, longer latency to fall asleep, and lower subjective sleep quality than men. As high‐quality sleep boosts physical and mental health, a better understanding of how stress, coping, and resilience are associated with sleep quality is vital to improving health outcomes, particularly during public health crises (Gentile et al., 2017; Hale et al., 2010; Milojevich & Lukowski, 2016; Peach et al., 2016; Tanaka & Shirakawa, 2004).

## 2 METHOD

### 2.1 Participants

Participants were a representative sample of adults living in the United States that matched the United States census by age, gender, and ethnicity. Participants were recruited from Prolific, an online recruitment platform (Prolific, 2020). Participants who were selected and interested in the study were directed to an online survey programmed on Qualtrics (Qualtrics, 2020) in English, which they completed between June 5 and 7, 2020. Due to the nature of the study's recruitment and administration, internet access and English literacy were two gateway requirements to participate. Participants were eligible for the survey if they were adults over the age of 18 that, at the time of the survey, lived in the United States. 400 participants completed the survey and, of those, 393 responded with complete data for all potential controls, predictor variables, and at least one sleep outcome, serving as the max analytic sample (*M*_age_ = 44.97; *SD*_age_ = 16.34; 50.5% women; 68% white; see Table 1 for full participant characteristics and demographics). For individual analyses, we made use of all available data using listwise exclusion, resulting in somewhat different sample sizes depending on the outcome variable of interest (see Table 1 for *n*s on individual variables).

**TABLE 1:** Participant demographics, stress, coping, resilience, and sleep descriptives in analytic sample

**Demographics and identity**					
**Variables**	**Categories or directionality**	***M* (SD) or % (*n*)**	**Observed range**	**Possible range**	***n***
Age		45.08 (16.37)	18–80		393
Gender	Women	50.9% (200)			393
	Men	49.1% (193)			
Employment	Unemployed, looking for work	9.9% (39)			393
	Other	90.1% (354)			
Subjective SES	Higher = Higher class	2.63 (0.84)	1–4		393
Highest degree	Higher = More educated	2.52 (1.10)	1–4		393
Race	White	67.7% (266)			389
	Black	14.0% (55)			
	Asian	7.6% (30)			
	Other	9.7% (38)			
**Coronavirus—Contextual**					
Objective infection rate in county^a^	Higher = Greater infection rate	679.64 (777.25)	20.61–4081.48		392
Objective mortality rate in county^a^	Higher = Greater mortality rate	44.93 (61.57)	0.73–261.81		375
**COVID‐19 stress and coping**					
Pandemic‐related stress	Higher = Greater pandemic‐related stress	2.35 (0.60)	1–3.8	1–4	393
Primary control engagement coping	Higher = Greater usage	20.79 (5.35)	9–36	9–36	393
Secondary control engagement coping	Higher = Greater usage	31.01 (6.48)	13–47	12–48	393
Disengagement coping	Higher = Greater usage	16.60 (5.12)	9–36	9–36	393
Involuntary engagement	Higher = Greater usage	25.61 (9.10)	15–59	15–60	393
Involuntary disengagement	Higher = Greater usage	19.24 (6.51)	12–41	12–48	393
**Brief Resilience Scale**					
Mean resilience	Higher = Greater resilience	3.39 (0.90)	1–5	1–5	393
**Pittsburgh Sleep Quality Index**					
Global sleep quality	Higher = Poorer sleep	6.61 (3.68)	0–17	0–21	355
Sleep latency	Higher = Longer latencies	1.29 (1.08)	0–3	0–3	389
Subjective sleep quality	Higher = Poorer quality	1.24 (0.68)	0–3	0–3	393

^a^ Variables were log‐transformed in all analyses, but raw values reported here to aid in interpretation.

### 2.2 Procedure

The survey opened with an informed consent section and concluded with a debrief that provided links to coronavirus and mental health resources. The survey also included an embedded attention‐check question, directing participants to “Select that you strongly disagree with this question” placed midway through the questionnaire within a block of questions that used a 5‐point scale ranging from *Strongly disagree* to *Strongly agree*. Participants who did not select *Strongly disagree* were coded as failing the attention‐check. Surveys included in the study were: the Responses to Stress Questionnaire (RSQ) (Connor‐Smith et al., 2000), the Brief Resilience Scale (BRS) (Smith et al., 2008), and the Pittsburgh Sleep Quality Index (PSQI) (Buysse et al., 1989). Participants were compensated for completing the survey at a rate of $9.50 per hour and the survey took 17 min on average to complete. All procedures were approved by the Institutional Review Board of Bryn Mawr College.

### 2.3 Measures

#### 2.3.1 Predictor variables

*Stress and coping strategies*: The RSQ assesses the ways that adults respond to and cope with stress (Connor‐Smith et al., 2000). The RSQ in this study was adapted to assess stress and coping specifically associated with COVID‐19 (used with permission from the Vanderbilt Stress and Coping Lab). The first section of this questionnaire lists 15 stressors pertaining to various aspects of COVID‐19 (financial problems, interpersonal challenges, practical difficulties due to quarantining, etc.) that prompted respondents to think about the magnitude of stress they have encountered during the spread of the novel coronavirus, coded as 1 = *Not at all* to 4 = *Very*. For example, participants indicated how stressful “Challenges at home or with others because of COVID‐19” have been. Responses across the 15 items were then averaged to produce a pandemic‐related stress score. The Cronbach's alpha reliability coefficient for this sample was 0.87.

The second section of the RSQ contains 57 items that ask participants to report the ways in which they responded to stressors during the pandemic. While items in this RSQ are tailored to COVID‐19, they stem from identical factors that contribute to various aspects of coping. The RSQ identifies five factors of coping (with example prompts): Primary Control Engagement Coping (“I try to think of ways to change or fix the situation”), Secondary Control Engagement Coping (“I realize that I just have to live with things the way they are”), Disengagement Coping (“I try not to think about it, to forget all about it”), Involuntary Engagement (“When I am dealing with the stress of COVID‐19, I feel it in my body”), and Involuntary Disengagement (“When faced with the stress of COVID‐19, I don't feel anything at all, it's like I have no feelings”) (Connor‐Smith et al., 2000). Participants responded on a scale from 1 = *Not at all* to 4 = *A lot* and responses for each subscale were averaged. The Cronbach's alpha reliability coefficients were: primary control engagement coping, 0.79; secondary control engagement coping, 0.81; disengagement coping, 0.77; involuntary engagement, 0.93; and involuntary disengagement, 0.88.

### 2.4 Resiliency

Participants responded to the BRS, a questionnaire that reliably assesses resilience as the ability to bounce back or recover from stress (Smith et al., 2008). BRS items ask respondents to indicate their agreement with six separate statements responding from 1 = *strongly disagree* to 5 = *strongly agree*. An example item from the inventory is “I tend to bounce back quickly after hard times.” The Cronbach's alpha reliability coefficient was 0.90 and the six items were averaged to produce a final BRS score.

#### 2.4.1 Outcome variables—Global sleep quality, sleep latency, and subjective sleep quality

Sleep measures were assessed using the PSQI, a questionnaire that assesses habitual sleep quality through self‐report. The global sleep quality score was calculated by summing all 7 component scores of the PSQI; scores ranged from 0 to 21, such that higher scores indicate poorer sleep (Buysse et al., 1989). The Cronbach's alpha reliability coefficient was 0.71. We also analyzed the sleep latency and subjective sleep quality components of the PSQI separately, as we were interested in determining whether gender disparities in these specific sleep components persisted during the coronavirus pandemic (Baker et al., 2007; Mallampalli & Carter, 2014; Ohayon et al., 2013). Participants’ responses to particular PSQI prompts were sometimes missing or uninterpretable, which resulted in lower participant numbers in the final scores (Table 1).

#### 2.4.2 Demographics

Participants reported their age, gender, highest level of education, current employment status, race and ethnicity, socioeconomic status, and ZIP code where they resided at the time of survey completion. Participants selected whether they identified as a woman (0), man (1) or other (2); four participants reported a gender that was not the gender binary category and, since this group was not adequately powered to draw meaningful conclusions about group differences, further analyses focused on participants who identified as men or women. Participants reported on their highest level of education, coded from 0 = some high school or only high school; 1 = Associate's degree or some college; 2 = Bachelor's Degree; or 3 = More than bachelor's degree. Participants reported whether they were currently unemployed and trying to find work (0 = Other; 1 = Unemployed, Looking for Work). To allow for reflexive self‐representation, participants reported their race and ethnicity using a “check‐all‐that‐apply” question that included: White, Hispanic, Black, Asian, Native Hawaiian or Pacific Islander, Prefer not to say, and Other (with a fill‐in option). We used participant responses to create any groups over 30 that emerged. Race and ethnicity was coded as 1 = White; 2 = Black; 3 = Asian; or 4 = Other (including multiracial/multiethnic participants who selected more than one). For subjective socioeconomic status, participants were asked to identify which social class they felt they belonged to on a 5‐point scale, such that higher scores indicated a higher subjective estimate of a participant's socioeconomic status (Dietze & Knowles, 2016).

Participants’ self‐reported resident zip codes were used to calculate objective coronavirus epidemiological infection and mortality rates for the county each participant was residing in at the time of the survey. These rates were derived from geographically based objective county infection counts collated by the Johns Hopkins University Center for Systems Science Dashboard (Dong et al., 2020). To create a *rate*, infection or mortality counts were divided by census population estimates for the respective county and multiplied by 100,000, resulting in an infection or mortality count per 100,000 person rate. Due to an extreme positive skew, raw values were log‐transformed, with a higher number representing higher infection and mortality rates. See Table 1 for the demographic breakdown and descriptives of this analytic sample.

### 2.5 Data analysis

For a linear regression with 10 predictors (our estimated max number of controls and predictors), an a priori power analysis was conducted in G*Power (Faul et al., 2009) and yielded a sample size of 254 with the following settings: power at 0.95, alpha error probability at .05, and a small to medium effect size (∼*f* ^2^ = 0.10); thus, our study was adequately powered.

To determine putative covariates for hypothesis testing, we examined whether patterns in bivariate relationships emerged between demographic variables and components of sleep at *p* < .05 and included variables that were associated with any sleep outcome across all three sleep hypothesis tests. Next, we examined general bivariate associations between predictor variables (gender, stress, coping, and resilience) and components of sleep, applying Pearson correlation, point‐biserial correlations, and ANOVA as appropriate (see Table 2). To determine the unique contribution of gender, stress, coping, and resilience to sleep components, hypothesis testing was conducted using linear regression, entering putative controls, stress, coping, and resilience simultaneously on a separate regression for each sleep component. We conducted data quality sensitivity analyses for each regression, repeating the regression while excluding the 19 participants who failed the attention‐check. In total, three separate regressions were run (one for each sleep measure), with three putative controls and seven predictor variables in each regression (see Table 3). All analyses were performed using SPSS Software version 26. In all analyses, α < 0.05 was regarded as statistically significant. To capitalize on all available data, analyses were conducted with available‐case analysis (pairwise deletion; see Tables 1 and 2 for individual construct *n*s).

**TABLE 2:** Bivariate associations of demographics, stress, coping, and resilience with sleep components

			Global sleep quality	Sleep latency	Subjective sleep quality
Demographics and coronavirus‐contextual controls	Age	*r*	**−0**.059	**−0.193**	**−0**.017
		*p*	.267	**<.001**	.738
		*n*	355	**389**	393
	Gender	*r*	**−0.152**	−0.083	**−0.105**
		*p*	**.004**	.101	**.037**
		*n*	**355**	389	**393**
	Race/ethnicity	*F*	1.385	2.485	2.336
		*p*	.247	.060	.073
		*n*	351	385	389
	Subjective SES	*r*	**−0.175**	−0.080	**−0.236**
		*p*	**.001**	.115	**<.0001**
		*n*	**355**	389	**393**
	Education level	*r*	−0.066	−0.094	−0.054
		*p*	.215	.064	.282
		*n*	355	389	393
	Unemployed, looking for work	*r*	−0.034	0.033	0.083
		*p*	.521	.513	.100
		*n*	355	389	393
	Objective infection rate	*r*	−0.060	−0.004	−0.024
		*p*	.260	.944	.638
		*n*	354	388	392
	Objective mortality rate	*r*	−0.061	−0004	−0.024
		*p*	.260	.944	.638
		*n*	339	372	375
Stress, coping, and resilience	Pandemic‐related stress	*r*	**0.307**	**0.257**	**0.277**
		*p*	**<.0001**	**<.0001**	**<.0001**
		*n*	**355**	**389**	**393**
	Primary control coping	*r*	0.030	−0.001	−0.053
		*p*	.574	.978	.294
		*n*	355	389	393
	Secondary control coping	*r*	**−0.170**	−0.081	**−0.209**
		*p*	**.001**	.112	**<.0001**
		*n*	**355**	389	**393**
	Disengagement coping	*r*	**0.192**	**0.156**	**0.157**
		*p*	**<.001**	**.002**	**.002**
		*n*	**355**	**389**	**393**
	Involuntary engagement	*r*	**0.432**	**0.288**	**0.361**
		*p*	**<.0001**	**<.0001**	**<.0001**
		*n*	**355**	**389**	**393**
	Involuntary disengagement	*r*	**0.381**	**0.259**	**0.297**
		*p*	**<.0001**	**<.0001**	**<.0001**
		*n*	**355**	**389**	**393**
	Brief Resilience Scale	*r*	**−0.394**	**−0.289**	**−0.326**
		*p*	**<.0001**	**<.0001**	**<.0001**
		*n*	**355**	**389**	**393**

*Notes*: **Bold** signifies significance at *p* < .05. Gender (0 = woman; 1 = man); Employment status (0 = Not unemployed, looking for work; 1 = Unemployed, looking for work). Continuous variables are coded such that higher is “more/greater” of the variable. PSQI variables are coded such that higher is “poorer/worse” of the variable. Race is a categorical variable and F‐tests from ANOVAs are reported.

**TABLE 3:** Parameter estimates, standard errors, and significance levels for regressions predicting global sleep quality, sleep latency, and subjective sleep quality

Variable		Global sleep quality	Sleep latency	Subjective sleep quality
*R* ^2^		0.283	0.155	0.231
*N*		355	389	393
Age	β	0.051	**−0.127**	0.049
	Std. Error	0.011	**0.003**	0.002
	*p*	.293	**.013**	.305
Gender	β	**−0.114**	−0.069	−0.075
	Std. Error	**0.348**	0.106	0.063
	*p*	**.017**	.160	.106
Subjective SES	β	−0.085	−0.020	**−0.155**
	Std. Error	0.206	0.063	**0.038**
	*p*	.072	.588	**.001**
Pandemic−related stress	β	0.098	0.124	**0.124**
	Std. Error	0.389	0.117	**0.070**
	*p*	.117	.056	**.043**
Primary control coping	β	**−0.138**	**−0.147**	**−0.166**
	Std. Error	**0.044**	**0.013**	**0.008**
	*p*	**.029**	**.024**	**.007**
Secondary control coping	β	0.039	0.088	**−0.028**
	Std. Error	0.036	0.011	0.006
	*p*	.523	.173	.649
Disengagement coping	β	**−0.183**	−0.107	−0.104
	Std. Error	**0.049**	0.015	0.009
	*p*	**.008**	.137	.124
Involuntary engagement	β	**0.318**	**0.205**	**0.316**
	Std. Error	**0.037**	**0.011**	**0.007**
	*p*	**<.001**	**.027**	**<.001**
Involuntary disengagement	β	0.141	0.029	0.013
	Std. Error	0.050	0.015	0.009
	*p*	.111	.752	.882
Brief Resilience Scale	β	**−0.199**	**−0.168**	**−0.114**
	Std. Error	**0.234**	**0.070**	**0.042**
	*p*	**<.001**	**.004**	**.041**

*Note*: **Bold** indicates that the relationships were significant (*p* < .05).

## 3 RESULTS

### 3.1 Preliminary analyses—Bivariate associations

Being older and being of higher subjective socioeconomic status were negatively associated with at least one sleep component (*p*s < 0.05) and were included as control variables in all hypotheses analyses (Table 2). Objective infection and mortality rates, education level, employment status, and race and ethnicity were not significantly associated with any component of sleep (*p*s > 0.05), and therefore were not included in further analyses (Table 2).

**FIGURE 1:**
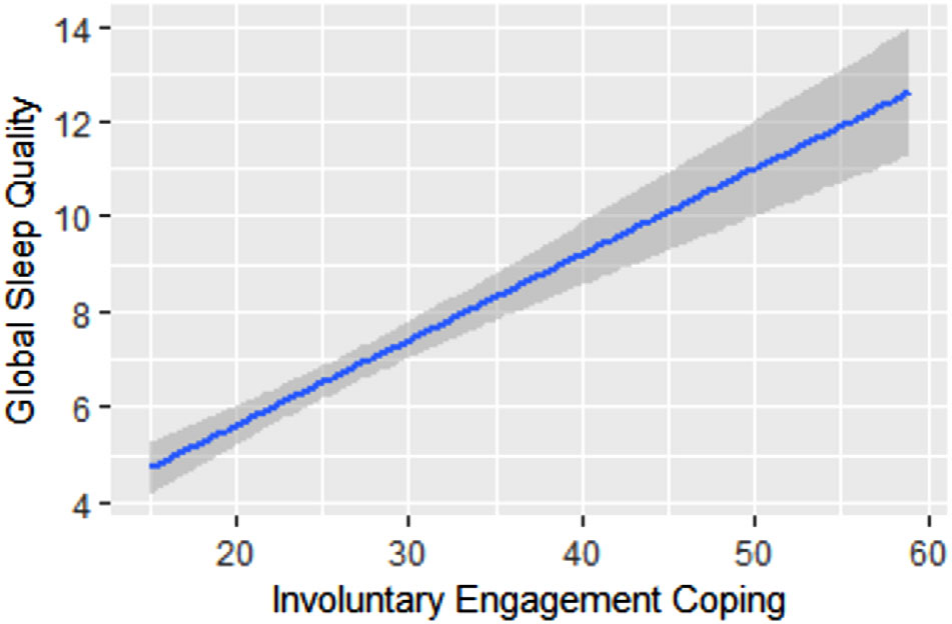
Bivariate relation between involuntary engagement coping and global sleep quality. *Note*. Involuntary engagement coping was coded such that higher numbers signify greater engagement in involuntary coping (measured via the Responses to Stress Questionnaire; Connor‐Smith et al., 2000) while global sleep quality was coded such that higher numbers signify worse global sleep quality (measured via the Pittsburgh Sleep Quality Index; Buysse et al., 1989).

Bivariate analyses revealed that women reported poorer global sleep quality than men (women: *M* = 7.15, *SD* = 3.97; men: *M* = 6.03, *SD* = 3.26, *t* = 2.91, *p* < .005). However, men and women did not significantly differ in their sleep latency score (*p* = .101). Additionally, women reported poorer subjective sleep quality than men (women: *M* = 1.31, SD = 0.70; men: *M* = 1.17, SD = 0.66, *t* = 2.10, *p* = .036). Resilience score and secondary control engagement coping were negatively associated with overall global sleep quality score (*p*s < 0.001; Table 2). Conversely, pandemic‐related stress, disengagement coping, and involuntary engagement and involuntary disengagement were positively associated with global sleep quality score (*p*s < 0.001; see Table 2 **;** see Figure 1 for data visualization of involuntary engagement coping and global sleep quality association).

### 3.2 Main analyses—Hypothesis testing

#### 3.2.1 Global sleep quality

Using linear regression, we first examined how gender, pandemic‐related stress, coping, and trait resilience were associated with global sleep quality scores in the PSQI while controlling for age and subjective social status. Identifying as a man (β = −0.114, *p* = .017), having greater resilience (β = −0.199, *p* < .001), and reporting greater use of primary control engagement (β = −0.138, *p* = .029) and disengagement coping (β = −0.183, *p* = .008) were associated significantly with better sleep quality (i.e., a lower global PSQI score). Greater involuntary engagement (β = 0.318, *p* < .001), on the other hand, was significantly associated with poorer sleep quality (i.e., a higher global PSQI score). Finally, pandemic‐related stress (β = 0.098, *p* = .117), secondary control engagement coping (β = 0.039, *p* = .523), and involuntary disengagement (β = 0.141, *p* = .111) were not significantly associated with global sleep quality scores. The overall model fit was *R* ^2^ = 0.283 (Table 3) and functional results (i.e., the direction and significance of the betas) persisted when the regression was rerun excluding participants who failed the attention‐check.

#### 3.2.2 Sleep latency

Greater resilience (β = −0.168, *p* = .004) and use of primary control engagement coping (β = −0.147, *p* = .024) were significantly associated with shorter latencies to falling asleep. On the other hand, greater involuntary engagement in response to pandemic‐related stress was associated with longer latencies to falling asleep (β = 0.205, *p* = .027). Finally, gender (β = −0.069, *p* = .160), pandemic‐related stress (β = 0.124, *p* = .056), secondary control engagement coping (β = 0.088, *p* = .173), disengagement coping (β = −0.107, *p* = .137) and involuntary disengagement (β = 0.029, *p* = .752) were not significantly associated with sleep latency while controlling for age and subjective SES. The overall model fit was *R* ^2^ = 0.155 (Table 3). When excluding those that failed the attention‐check, the relation between greater involuntary engagement and longer latency to falling asleep remained positive, but was trending and failed to reach significance (*p* = .061); otherwise, all functional results persisted.

#### 3.2.3 Subjective sleep quality

Reporting lower pandemic‐related stress (β = 0.124, *p* = .043), greater resilience (β = −0.114, *p* = .041), more primary control engagement coping (β = −0.166, *p* = .007), and less involuntary engagement (β = 0.316, *p* < .001) were significantly associated with better subjective sleep quality while controlling for age and subjective SES. Gender (β = −0.075, *p* = .106), secondary control engagement coping (β = −0.028, *p* = .649), disengagement coping (β = −0.104, *p* = .124), and involuntary disengagement (β = 0.013, *p* = .882) were not significantly associated with subjective sleep quality. The overall model fit was *R* ^2^ = .231 (Table 3) and functional results were the same when the regression was rerun excluding participants who failed the attention‐check.

## 4 DISCUSSION

The purpose of this study was to examine factors that affect sleep quality during the coronavirus pandemic. Specifically, we were interested in determining if factors such as pandemic‐related stress, particular coping strategies, and resilience were associated with sleep quality while addressing relevant demographic and coronavirus‐contextual controls. Interestingly, pandemic‐related stress was not significantly associated with sleep quality, and current research examining this relationship, particularly in US adult civilians, remains unclear (Benham, 2020; Kim‐Godwin et al., 2021; Tsang et al., 2021; Ulrich et al., 2021). Our results instead suggest that resilience and coping during the coronavirus pandemic may influence sleep quality more than pandemic‐related stress, which presents interesting applied implications. Involuntary engagement coping, which includes rumination and physiological arousal, was associated with poor sleep quality. In contrast, greater primary control engagement coping and resilience were associated with better sleep quality. In addition, consistent with past research, gender was associated with sleep quality, such that women experienced worse sleep quality compared with men during the COVID‐19 pandemic (Cellini et al., 2021; Foster et al., 2017; Franceschini et al., 2020; Hajali et al., 2019; Mong & Cusmano, 2016; Paiva et al., 2021; Salfi et al., 2020; Siddique et al., 2021). Thus, the results of our study confirm prior research to demonstrate that gender disparities in sleep persist during pandemics while addressing robust sociodemographic and contextual controls. In addition, our study is the first to establish that coping and resilience are more strongly associated with sleep quality than stress or local infection rates during a pandemic.

Previous studies showed associations between stress and sleep during COVID‐19 (Ahmed et al., 2021; Alimoradi et al., 2021; Benham, 2020; Browning et al., 2021; Guo et al., 2020; Kim‐Godwin et al., 2021; Tsang et al., 2021; Ulrich et al., 2021; Wright et al., 2020) and the present results may diverge from these findings for a few reasons. First, the present study examined pandemic‐related stress, while the majority of research on coronavirus assessed stress using general stress measures, which precludes conclusions about the influence of the stress of COVID‐19 on sleep. Next, though we hypothesized that pandemic‐related stress would be associated with worse sleep quality, an adjusted, bivariate negative relationship between pandemic‐related stress and sleep quality was not sustained when accounting for robust demographic, contextual, coping, and resilience‐based factors. Interestingly, pandemic‐related stress was influenced by the outbreak as there was a positive association between objective infection rates and pandemic‐related stress (Table S1). However, pandemic‐related stress was not a significant factor in sleep health. These findings were surprising and suggest that alternative factors are more strongly associated with sleep than pandemic‐related stress, or perhaps even fully mediate the impact of pandemic‐related stress upon sleep, which is a direction for future research.

Since coping strategies may directly influence sleep quality, they are important to be considered (El‐Sheikh et al., 2014; Grafe et al., 2020; Pillai et al., 2014; Tada, 2017; Zhang et al., 2020). Our study indicated that a maladaptive coping strategy, namely, involuntary engagement, was associated with poorer global sleep quality. Involuntary engagement includes components such as physiological arousal and rumination, which have been shown in previous studies (outside of pandemic conditions) to be associated with impaired sleep (Y. Li et al., 2019; Tousignant et al., 2019; Zoccola et al., 2009). Stress‐induced physiological arousal includes sympathetic activity, which prevents the decrease in blood pressure normally observed in slow‐wave sleep, ultimately decreasing sleep quality (Seravalle et al., 2018; Sherwood et al., 2011). Moreover, when an individual ruminates, they tend to repeatedly think about or fixate on a situation (Lazarus, 2006), which has been associated with insomnia and poor sleep quality (Pillai et al., 2014). The present findings also align with recent COVID‐19 specific research, showing that involuntary engagement, particularly through rumination, reduced sleep quality in college students during the pandemic (Du et al., 2020). Our study supports and extends these findings to a representative sample of US adults. Furthermore, our results demonstrate that involuntary engagement coping is more strongly associated with sleep quality than pandemic‐related stress or objective infection rates. Beyond global sleep quality, involuntary engagement coping was also associated with longer latencies to fall asleep and poorer subjective sleep quality. In sum, while involuntary engagement in response to acute stressors can be adaptive in the short‐term (McEwen, 2000), high involuntary engagement during a chronic stressor, such as the pandemic, relates to poorer sleep measures.

In contrast, we found that adaptive coping strategies, such as primary control engagement coping, which focuses on directly altering the stressor or one's response to it, were associated with better global sleep quality. Moreover, this adaptive coping strategy was associated with shorter latencies to falling asleep and greater subjective sleep quality. For some time now, adaptive coping strategies included in primary control engagement coping, such as problem solving and emotional regulation, have been associated with better quality sleep (C. Chen, 2016; H.‐M. Chen et al., 2015; Wood et al., 2010). Our study demonstrates that this coping strategy is also associated with better quality sleep in the context of a pandemic. In contrast, we found that other adaptive coping strategies, including secondary control coping, which is focused on adaptation to a stressor and includes components such as acceptance and positive thinking (Connor‐Smith et al., 2000), were not associated with sleep quality. These findings indicate that active steps taken to alleviate pandemic‐related stress and regulate emotions towards the stress are associated with better sleep (more so than attempts to adapt to the stress). Interestingly, these COVID‐19‐related coping strategies were associated with sleep outcomes over and above the positive relation between general resilience and healthy sleep, suggesting that enhancing pandemic‐specific coping has benefits across people who are higher and lower in general resilience. Enhancing positive coping during pandemics could be a fruitful intervention to improve sleep even among those who may be psychologically vulnerable. The results contribute to a growing body of research suggesting that certain adaptive coping strategies are more effective in protecting against impaired sleep during stressful circumstances like the current COVID‐19 pandemic.

Our results indicate that trait resilience, which describes the ability to "bounce back," recover from, or endure a stressor, was associated with better sleep quality. Like primary control engagement coping, greater resilience was associated with better global sleep quality, shorter latencies to sleeping, and better subjective sleep quality. As a stable trait (Smith et al., 2008), resilience is an important factor in reducing stress and its effects (C. Chen, 2016; Y. Li et al., 2019; McCanlies et al., 2014; Wood et al., 2010). Importantly, low resilience is related to a dysregulation in the stress response and persistent psychopathology, in which sleep disturbances are common (Hjemdal et al., 2011; Wu et al., 2013). Moreover, previous studies suggest that high resilience is associated with better sleep (Palagini et al., 2018; Seelig et al., 2016). Thus, the present results are consistent with previous findings suggesting that resilience is associated with positive mental health outcomes including better sleep, even during the coronavirus pandemic.

Consistent with previous research (Cellini et al., 2021; Franceschini et al., 2020; Liang et al., 2020; Mohammadzadeh et al., 2020; Paiva et al., 2021; Siddique et al., 2021; Xiong et al., 2020), we found that women reported poorer global sleep quality compared to men during the coronavirus pandemic. Further, our study extended these findings by demonstrating that these gender disparities persisted even when accounting for subjective socioeconomic status, stress, coping, and other potentially relevant covariates. However, though the previous literature indicates that women report a longer latency to fall asleep and lower subjective sleep quality than men in non‐pandemic conditions (Baker et al., 2007; Mallampalli & Carter, 2014; Ohayon et al., 2013), this disparity did not persist in our representative sample of US adults during the coronavirus pandemic. In contrast to our research, other studies found increased sleep latency in women compared to men during the COVID‐19 pandemic (Casagrande et al., 2020; Del Río‐Casanova et al., 2021; Liang et al., 2020). However, these populations were from countries with much higher COVID‐19 infection rates at the time of data collection, which could affect sleep latency scores. In addition, we were surprised that our results indicated that subjective sleep quality did not differ between genders (because global sleep quality scores did show gender disparities); however, it is important to note the differences between subjective sleep quality and global sleep quality scores. In short, the PSQI global sleep quality score consists of several components including, but not limited to: sleep duration, sleep efficiency, sleep disturbances, and daytime dysfunction. In contrast, the subjective sleep quality component only contains one item, in which participants rate their sleep in the past month. While this PSQI item may not be the ideal representation of subjective sleep quality, the construct of subjective sleep quality remains elusive and lacks a clear definition in the literature (Conte et al., 2020; Goelema et al., 2018).

Insights from animal models suggest that gonadal hormones such as estrogen may partially drive sex differences in sleep responses to stress (Gargiulo et al., 2021; Grafe et al., 2020; Mong & Cusmano, 2016; Walker et al., 2019). However, future research on pandemics should continue to address further gender disparate factors (e.g., sexism, childrearing and caretaking responsibilities, sleep disorder and stress‐related psychiatric disorder incidence, etc.) that may contribute to gender disparities in sleep during pandemics (Kessler et al., 1994; Majeno et al., 2018; Mong & Cusmano, 2016; Pepin et al., 2018; Slopen et al., 2016). In addition, future research on sleep quality during the COVID‐19 pandemic could enhance recruitment of transgender and non‐binary people to ensure adequately powered analyses across gender diversity (e.g., see Rodriguez‐Seijas et al., 2020 for a study on psychological outcomes during COVID‐19).

This study contributes to the rapidly developing literature emerging on the psychosocial impact of the novel coronavirus. Our study was highly powered and representative of the United States on age, race, and gender. Moreover, our analyses accounted for both demographic factors (e.g., age, subjective SES) and coronavirus‐contextual factors (e.g., being unemployed and looking for work, local epidemiological infection rates) that could be associated with both hypothesized predictors and sleep outcomes, strengthening the internal and external validity of the findings.

Despite the strengths of our study, there are aspects of sample representation, historical context, and design worth considering as limitations. While the sample matched the United States population on age, gender, and race, our survey was only offered in English. Survey language accessibility is essential in future research to ensure the representation of marginalized groups and inclusion of Spanish speakers in the United States (Ortega et al., 2020). Moreover, it is important to note that the stress of COVID‐19 does not occur in a vacuum and the data collection period coincided with widespread national protests in response to George Floyd's murder (Buchanan et al., 2020). Other research illustrates that the “dual pandemics” of racial oppression and COVID‐19 were deeply stressful during COVID‐19, particularly for people of color (Molock & Parchem, 2021; Trammell et al., 2021). Future research on COVID‐19, stress, and sleep should include discriminatory aspects of COVID‐19 in stress measures alongside assessments of racial trauma (Williams et al., 2018).

Importantly, this study only provides a snapshot of individuals’ stress during the coronavirus pandemic. The data were collected in June of 2020 during a particularly telling time in the pandemic, with widespread restrictions, financial strain, and job loss, yet relief legislation was unevenly distributed at the state and federal levels. Despite the meaningful historical moment captured in the present study, longitudinal research has demonstrated that population stress levels related to the pandemic fluctuate, with indicators for anxiety or depressive disorders rising steadily across 2020 (*Mental Health ‐ Household Pulse Survey ‐ COVID‐19*, 2021). Thus, longitudinal data would provide more information about the stability of stress, coping, and sleep across the time course of the coronavirus pandemic and would enable disaggregation of directionality effects between coping and sleep. In addition, longitudinal research would allow further investigation of mechanisms by which resilience and coping interact with stress and sleep. Finally, sleep was operationalized using self‐report. While the PSQI is a clinically relevant indicator of sleep, past research has shown divergent results between self‐report and actigraphy‐assessed sleep (Peterson et al., 2017), and future research should seek to validate the present results using more objective techniques. Wearable actigraphy monitors would allow for a more objective, but simultaneously ecological assessment of sleep.

The adverse mental health consequences of the COVID‐19 pandemic are apparent, with the need for greater psychosocial intervention and care for the public (Pfefferbaum & North, 2020). This study sought to better understand the associations between pandemic‐related stress, coping, resilience, and sleep quality, particularly in men and women living in the United States. Findings demonstrate the importance of enhancing adaptive strategies (like primary control engagement coping) while attenuating maladaptive strategies (like involuntary engagement coping) in order to promote sleep health in the face of major stressors like the coronavirus pandemic. Adaptive coping is malleable and a target for interventions; cognitive behavioral therapy (CBT) and mindfulness‐based stress reduction (MBSR) interventions increase adaptive coping strategies such as emotional regulation and reduce maladaptive coping strategies such as rumination (Berghmans et al., 2012; Chiesa & Serretti, 2009; Friedrich & Schlarb, 2018; Long et al., 2021; Shatkin et al., 2016). Moreover, both CBT and MBSR interventions enhance sleep quality in non‐pandemic conditions (T.‐L. Chen et al., 2020; Ramsawh et al., 2016). Thus, CBT and MBSR could potentially be utilized in global health emergencies to improve coping skills and ultimately, sleep quality, and is a direction for future research. The present results provide a fruitful foundation, demonstrating that enhanced pandemic coping associates with healthier sleep across racial groups, socioeconomic status, regionalized virus severity, pandemic‐stress levels, and over and above general resilience. Healthy sleep is critical to strong immune function, mental health, and cognition, and a better understanding of how stress, coping, and resilience are associated with sleep quality is vital to improving health outcomes during public health crises.

## CONFLICT OF INTEREST

The authors declare no conflict of interest.

## DATA ACCESSIBILITY STATEMENT

The data that support the findings of this study are available on request from the corresponding author. The data are not publicly available due to privacy or ethical restrictions.

### PEER REVIEW

The peer review history for this article is available at https://publons.com/publon/10.1002/brb3.2384

## Supporting information

Supporting Information

## References

[brb32384-bib-0001] Ahmed, O. , Hossain, K. N. , Siddique, R. F. , & Jobe, M. C. (2021). COVID‐19 fear, stress, sleep quality and coping activities during lockdown, and personality traits: A person‐centered approach analysis. Personality and Individual Differences, 178, 110873. 10.1016/j.paid.2021.110873 PMC9755889 36540788

[brb32384-bib-0002] Al Maqbali, M. , & Al Khadhuri, J. (2021). Psychological impact of the coronavirus 2019 (COVID‐19) pandemic on nurses. Japan Journal of Nursing Science, 18, e12417. 10.1111/jjns.12417 PMC8250093 33749144

[brb32384-bib-0003] Alimoradi, Z. , Broström, A. , Tsang, H. W. H. , Griffiths, M. D. , Haghayegh, S. , Ohayon, M. M. , Lin, C.‐Y. , & Pakpour, A. H. (2021). Sleep problems during COVID‐19 pandemic and its’ association to psychological distress: A systematic review and meta‐analysis. EClinicalMedicine, 36, 100916. 10.1016/j.eclinm.2021.100916 34131640 PMC8192091

[brb32384-bib-0004] Baker, F. C. , Kahan, T. L. , Trinder, J. , & Colrain, I. M. (2007). Sleep quality and the sleep electroencephalogram in women with severe premenstrual syndrome. Sleep, 30(10), 1283–1291. 17969462 10.1093/sleep/30.10.1283 PMC2266284

[brb32384-bib-0005] Benca, R. M. (1996). Sleep in psychiatric disorders. Neurologic Clinics, 14(4), 739–764. 8923493 10.1016/s0733-8619(05)70283-8

[brb32384-bib-0006] Benham, G. (2020). Stress and sleep in college students prior to and during the COVID‐19 pandemic. Stress and Health, 37(3), 504–515. 10.1002/smi.3016 33315301

[brb32384-bib-0007] Berghmans, C. , Godard, R. , Joly, J. , Tarquinio, C. , & Cuny, P. (2012). Effects of the mindfulness based stress reduction (MBSR) approach on psychic health (stress, anxiety, depression) and coping mode of diabetic patients: A controlled and randomized pilot study. Annales Medico‐Psychologiques, 170(5), 312–317. 10.1016/j.amp.2010.08.010

[brb32384-bib-0008] Blume, C. , Schmidt, M. H. , & Cajochen, C. (2020). Effects of the COVID‐19 lockdown on human sleep and rest‐activity rhythms. Current Biology, 30(14), R795–R797. 10.1016/j.cub.2020.06.021 32693067 PMC7284244

[brb32384-bib-0009] Breslau, N. , Roth, T. , Burduvali, E. , Kapke, A. , Schultz, L. , & Roehrs, T. (2004). Sleep in lifetime posttraumatic stress disorder. Archives of General Psychiatry, 61(5), 508. 10.1001/archpsyc.61.5.508 15123496

[brb32384-bib-0010] Brooks, S. K. , Webster, R. K. , Smith, L. E. , Woodland, L. , Wessely, S. , Greenberg, N. , & Rubin, G. J. (2020). The psychological impact of quarantine and how to reduce it: Rapid review of the evidence. Lancet, 395(10227), 912–920. 10.1016/S0140-6736(20)30460-8 32112714 PMC7158942

[brb32384-bib-0011] Browning, M. H. E. M. , Larson, L. R. , Sharaievska, I. , Rigolon, A. , McAnirlin, O. , Mullenbach, L. , Cloutier, S. , Vu, T. M. , Thomsen, J. , Reigner, N. , Metcalf, E. C. , D'Antonio, A. , Helbich, M. , Bratman, G. N. , & Alvarez, H. O. (2021). Psychological impacts from COVID‐19 among university students: Risk factors across seven states in the United States. PLOS ONE, 16(1), e0245327. 10.1371/journal.pone.0245327 33411812 PMC7790395

[brb32384-bib-0012] Buchanan, L. , Parlapiano, A. , Parshina‐Kottas, Y. , Patanjali, K. , Saget, B. , Singhvi, A. , Wu, J. , & Yourish, K. (2020). Bird's eye view of protests across the U.S. and around the world. The New York Times. http://www.nytimes.com/interactive/2020/06/07/us/george-floyd-protest-aerial-photos.html

[brb32384-bib-0013] Buysse, D. J. , Reynolds, C. F. , Monk, T. H. , Berman, S. R. , & Kupfer, D. J. (1989). The Pittsburgh Sleep Quality Index: A new instrument for psychiatric practice and research. Psychiatry Research, 28(2), 193–213. 10.1016/0165-1781(89)90047-4 2748771

[brb32384-bib-0014] Carney, C. E. , Edinger, J. D. , Meyer, B. , Lindman, L. , & Istre, T. (2006). Symptom‐focused rumination and sleep disturbance. Behavioral Sleep Medicine, 4(4), 228–241. 10.1207/s15402010bsm0404_3 17083303

[brb32384-bib-0015] Carver , C. S. , Scheier, M. F. , & Weintraub, J. K. (1989). Assessing coping strategies: A theoretically based approach. Journal of Personality and Social Psychology, 56(2), 267–283. 2926629 10.1037//0022-3514.56.2.267

[brb32384-bib-0016] Casagrande, M. , Favieri, F. , Tambelli, R. , & Forte, G. (2020). The enemy who sealed the world: Effects quarantine due to the COVID‐19 on sleep quality, anxiety, and psychological distress in the Italian population. Sleep Medicine, 75, 12–20. 10.1016/j.sleep.2020.05.011 32853913 PMC7215153

[brb32384-bib-0017] Cellini, N. , Conte, F. , De Rosa, O. , Giganti, F. , Malloggi, S. , Reyt, M. , Guillemin, C. , Schmidt, C. , Muto, V. , & Ficca, G. (2021). Changes in sleep timing and subjective sleep quality during the COVID‐19 lockdown in Italy and Belgium: Age, gender and working status as modulating factors. Sleep Medicine, 77, 112–119. 10.1016/j.sleep.2020.11.027 33348298 PMC9183798

[brb32384-bib-0018] Chang, L.‐Y. , Wu, C.‐C. , Lin, L. N. , Chang, H.‐Y. , & Yen, L.‐L. (2019). Age and sex differences in the effects of peer victimization on depressive symptoms: Exploring sleep problems as a mediator. Journal of Affective Disorders, 245, 553–560. 10.1016/j.jad.2018.11.027 30439680

[brb32384-bib-0019] Chen, C. (2016). The role of resilience and coping styles in subjective well‐being among Chinese university students. The Asia‐Pacific Education Researcher, 25(3), 377–387. 10.1007/s40299-016-0274-5

[brb32384-bib-0020] Chen, H.‐M. , Huang, M.‐F. , Yeh, Y.‐C. , Huang, W.‐H. , & Chen, C.‐S. (2015). Effectiveness of coping strategies intervention on caregiver burden among caregivers of elderly patients with dementia. Psychogeriatrics, 15(1), 20–25. 10.1111/psyg.12071 25515800

[brb32384-bib-0021] Chen, T.‐L. , Chang, S.‐C. , Hsieh, H.‐F. , Huang, C.‐Y. , Chuang, J.‐H. , & Wang, H.‐H. (2020). Effects of mindfulness‐based stress reduction on sleep quality and mental health for insomnia patients: A meta‐analysis. Journal of Psychosomatic Research, 135, 110144. 10.1016/j.jpsychores.2020.110144 32590218

[brb32384-bib-0022] Chiesa, A. , & Serretti, A. (2009). Mindfulness‐based stress reduction for stress management in healthy people: A review and meta‐analysis. The Journal of Alternative and Complementary Medicine, 15(5), 593–600. 10.1089/acm.2008.0495 19432513

[brb32384-bib-0023] Chu, I. Y.‐H. , Alam, P. , Larson, H. J. , & Lin, L. (2020). Social consequences of mass quarantine during epidemics: A systematic review with implications for the COVID‐19 response. Journal of Travel Medicine, 27(7). 10.1093/jtm/taaa192 PMC7649384 33051660

[brb32384-bib-0024] Compas, B. E. , Connor, J. , Osowiecki, D. , & Welch, A. (1997). Effortful and involuntary responses to stress. In B. H. Gottlieb (Ed.), Coping with chronic stress (pp. 105–130). Springer US. 10.1007/978-1-4757-9862-3_4

[brb32384-bib-0025] Compas, B. E. , Jaser, S. S. , Bettis, A. H. , Watson, K. H. , Gruhn, M. , Dunbar, J. P. , Williams, E. , & Thigpen, J. C. (2017). Coping, emotion regulation and psychopathology in childhood and adolescence: A meta‐analysis and narrative review. Psychological Bulletin, 143(9), 939–991. 10.1037/bul0000110 28616996 PMC7310319

[brb32384-bib-0026] Conklin, L. R. , Cassiello‐Robbins, C. , Brake, C. A. , Sauer‐Zavala, S. , Farchione, T. J. , Ciraulo, D. A. , & Barlow, D. H. (2015). Relationships among adaptive and maladaptive emotion regulation strategies and psychopathology during the treatment of comorbid anxiety and alcohol use disorders. Behaviour Research and Therapy, 73, 124–130. 10.1016/j.brat.2015.08.001 26310363 PMC4573351

[brb32384-bib-0027] Connor‐Smith, J. K. , Compas, B. E. , Wadsworth, M. E. , Thomsen, A. H. , & Saltzman, H. (2000). Responses to stress in adolescence: Measurement of coping and involuntary stress responses. Journal of Consulting and Clinical Psychology, 68(6), 976–992. 10.1037/0022-006X.68.6.976 11142550

[brb32384-bib-0028] Conte, F. , Cerasuolo, M. , Fusco, G. , Giganti, F. , Inserra, I. , Malloggi, S. , Di Iorio, I. , & Ficca, G. (2020). Sleep continuity, stability and organization in good and bad sleepers. Journal of Health Psychology, 26, 2131–2142. 10.1177/1359105320903098 32031019

[brb32384-bib-0029] Dahlgren, A. , Kecklund, G. , & Åkerstedt, T. (2005). Different levels of work‐related stress and the effects on sleep, fatigue and cortisol. Scandinavian Journal of Work, Environment & Health, 31(4), 277–285. 10.5271/sjweh.883 16161710

[brb32384-bib-0030] Del Río‐Casanova, L. , Sánchez‐Martín, M. , García‐Dantas, A. , González‐Vázquez, A. , & Justo, A. (2021). Psychological responses according to gender during the early stage of COVID‐19 in Spain. International Journal of Environmental Research and Public Health, 18(7), 3731. 10.3390/ijerph18073731 33918378 PMC8038227

[brb32384-bib-0032] Dhama, K. , Khan, S. , Tiwari, R. , Sircar, S. , Bhat, S. , Malik, Y. S. , Singh, K. P. , Chaicumpa, W. , Bonilla‐Aldana, D. K. , & Rodriguez‐Morales, A. J. (2020). Coronavirus disease 2019–COVID‐19. Clinical Microbiology Reviews, 33(4). 10.1128/CMR.00028-20 PMC7405836 32580969

[brb32384-bib-0033] Dietze, P. , & Knowles, E. (2016). Social class and the motivational relevance of other human beings. Psychological Science, 27. 10.1177/0956797616667721 27698091

[brb32384-bib-0034] Dong, E. , Du, H. , & Gardner, L. (2020). An interactive web‐based dashboard to track COVID‐19 in real time. The Lancet Infectious Diseases, 20, 533–534. 10.1016/S1473-3099(20)30120-1 32087114 PMC7159018

[brb32384-bib-0035] Du, C. , Zan, M. C. H. , Cho, M. J. , Fenton, J. I. , Hsiao, P. Y. , Hsiao, R. , Keaver, L. , Lai, C.‐C. , Lee, H. , Ludy, M.‐J. , Shen, W. , Swee, W. C. S. , Thrivikraman, J. , Tseng, K.‐W. , Tseng, W.‐C. , & Tucker, R. M. (2020). Increased resilience weakens the relationship between perceived stress and anxiety on sleep quality: A moderated mediation analysis of higher education students from 7 countries. Clocks & Sleep, 2(3), 334–353. 10.3390/clockssleep2030025 33089208 PMC7573806

[brb32384-bib-0036] Eder, S. J. , Steyrl, D. , Stefanczyk, M. M. , Pieniak, M. , Molina, J. M. , Pešout, O. , Binter, J. , Smela, P. , Scharnowski, F. , & Nicholson, A. A. (2021). Predicting fear and perceived health during the COVID‐19 pandemic using machine learning: A cross‐national longitudinal study. PLOS ONE, 16(3), e0247997. 10.1371/journal.pone.0247997 33705439 PMC7951840

[brb32384-bib-0037] El‐Sheikh, M. , Kelly, R. J. , Sadeh, A. , & Buckhalt, J. A. (2014). Income, ethnicity and sleep: Coping as a moderator. Cultural Diversity & Ethnic Minority Psychology, 20(3), 441–448. 10.1037/a0036699 25045954 PMC4105988

[brb32384-bib-0038] Faul, F. , Erdfelder, E. , Axel, B. , & Albert‐George, L. (2009). Statistical power analyses using G*Power 3.1: Tests for correlation and regression analyses. Behavior Research Methods. 10.3758/BRM.41.4.1149 19897823

[brb32384-bib-0039] Finnell, J. E. , Lombard, C. M. , Melson, M. N. , Singh, N. P. , Nagarkatti, M. , Nagarkatti, P. , Fadel, J. R. , Wood, C. S. , & Wood, S. K. (2017). The protective effects of resveratrol on social stress‐induced cytokine release and depressive‐like behavior. Brain, Behavior, and Immunity, 59, 147–157. 10.1016/j.bbi.2016.08.019 PMC5154920 27592314

[brb32384-bib-0040] Folkman, S. , & Moskowitz, J. T. (2004). Coping: Pitfalls and promise. Annual Review of Psychology, 55(1), 745–774. 10.1146/annurev.psych.55.090902.141456 14744233

[brb32384-bib-0041] Foster, S. N. , Hansen, S. L. , Capener, D. C. , Matsangas, P. , & Mysliwiec, V. (2017). Gender differences in sleep disorders in the US military. Sleep Health, 3(5), 336–341. 10.1016/j.sleh.2017.07.015 28923189

[brb32384-bib-0042] Franceschini, C. , Musetti, A. , Zenesini, C. , Palagini, L. , Scarpelli, S. , Quattropani, M. C. , Lenzo, V. , Freda, M. F. , Lemmo, D. , Vegni, E. , Borghi, L. , Saita, E. , Cattivelli, R. , De Gennaro, L. , Plazzi, G. , Riemann, D. , & Castelnuovo, G. (2020). Poor sleep quality and its consequences on mental health during the COVID‐19 lockdown in Italy. Frontiers in Psychology, 11, 574475. 10.3389/fpsyg.2020.574475 33304294 PMC7693628

[brb32384-bib-0043] Friedrich, A. , & Schlarb, A. A. (2018). Let's talk about sleep: A systematic review of psychological interventions to improve sleep in college students. Journal of Sleep Research, 27(1), 4–22. 10.1111/jsr.12568 28618185

[brb32384-bib-0044] Gargiulo, A. T. , Jasodanand, V. , Luz, S. , O'Mara, L. , Kubin, L. , Ross, R. J. , Bhatnagar, S. , & Grafe, L. A. (2021). Sex differences in stress‐induced sleep deficits. Stress, 24, 541–550. 10.1080/10253890.2021.1879788 33525935

[brb32384-bib-0045] Gas, S. , Eksi Ozsoy, H. , & Cesur Aydin, K. (2021). The association between sleep quality, depression, anxiety and stress levels, and temporomandibular joint disorders among Turkish dental students during the COVID‐19 pandemic. Cranio: The Journal of Craniomandibular & Sleep Practice. 10.1080/08869634.2021.1883364 33543679

[brb32384-bib-0046] Gentile, A. R. , Sylvester, B. D. , & Sabiston, C. M. (2017). Be active, rest well, and improve mental health: Physical activity, depression symptoms and the mediating role of sleep quality and quantity among breast cancer survivors. Journal of Sport & Exercise Psychology, 39, S253–S253.

[brb32384-bib-0047] Goelema, M. , Leufkens, T. , Haakma, R. , & Markopoulos, P. (2018). Determinants of self‐reported sleep quality in healthy sleepers and patients. Cogent Psychology, 5(1), 1499197. 10.1080/23311908.2018.1499197

[brb32384-bib-0048] Grafe, L. A. , O'Mara, L. , Branch, A. , Dobkin, J. , Luz, S. , Vigderman, A. , Shingala, A. , Kubin, L. , Ross, R. , & Bhatnagar, S. (2020). Passive coping strategies during repeated social defeat are associated with long‐lasting changes in sleep in rats. Frontiers in Systems Neuroscience, 14, 6. 10.3389/fnsys.2020.00006 32140101 PMC7043017

[brb32384-bib-0049] Griffin, R. J. , Dunwoody, S. , & Zabala, F. (1998). Public reliance on risk communication channels in the wake of a cryptosporidium outbreak. Risk Analysis, 18(4), 367–375. 10.1111/j.1539-6924.1998.tb00350.x 9775446

[brb32384-bib-0050] Guo, J. , Feng, X. L. , Wang, X. H. , & van IJzendoorn, M. H. (2020). Coping with COVID‐19: Exposure to COVID‐19 and negative impact on livelihood predict elevated mental health problems in Chinese adults. International Journal of Environmental Research and Public Health, 17(11), 3857. 10.3390/ijerph17113857 PMC7312167 32485859

[brb32384-bib-0051] Hajali, V. , Andersen, M. L. , Negah, S. S. , & Sheibani, V. (2019). Sex differences in sleep and sleep loss‐induced cognitive deficits: The influence of gonadal hormones. Hormones and Behavior, 108, 50–61. 10.1016/j.yhbeh.2018.12.013 30597139

[brb32384-bib-0052] Hale, L. , Hill, T. D. , & Burdette, A. M. (2010). Does sleep quality mediate the association between neighborhood disorder and self‐rated physical health? Preventive Medicine, 51(3–4), 275–278. 10.1016/j.ypmed.2010.06.017 20600254

[brb32384-bib-0053] Held, P. , Owens, G. P. , Schumm, J. A. , Chard, K. M. , & Hansel, J. E. (2011). Disengagement coping as a mediator between trauma‐related guilt and PTSD severity. Journal of Traumatic Stress, 24(6), 708–715. 10.1002/jts.20689 22131291

[brb32384-bib-0054] Hjemdal, O. , Vogel, P. A. , Solem, S. , Hagen, K. , & Stiles, T. C. (2011). The relationship between resilience and levels of anxiety, depression, and obsessive‐compulsive symptoms in adolescents. Clinical Psychology & Psychotherapy, 18(4), 314–321. 10.1002/cpp.719 20806419

[brb32384-bib-0055] Hoge, E. A. , Marques, L. , Wechsler, R. S. , Lasky, A. K. , Delong, H. R. , Jacoby, R. J. , Worthington, J. J. , Pollack, M. H. , & Simon, N. M. (2011). The role of anxiety sensitivity in sleep disturbance in panic disorder. Journal of Anxiety Disorders, 25(4), 536–538. 10.1016/j.janxdis.2010.12.008 21277737

[brb32384-bib-0056] Jahrami, H. , BaHammam, A. S. , AlGahtani, H. , Ebrahim, A. , Faris, M. , AlEid, K. , Saif, Z. , Haji, E. , Dhahi, A. , Marzooq, H. , Hubail, S. , & Hasan, Z. (2020). The examination of sleep quality for frontline healthcare workers during the outbreak of COVID‐19. Sleep and Breathing, 25, 503–511. 10.1007/s11325-020-02135-9 32592021 PMC7319604

[brb32384-bib-0057] Kessler, R. C. , McGonagle, K. A. , Nelson, C. B. , Hughes, M. , Swartz, M. , & Blazer, D. G. (1994). Sex and depression in the National Comorbidity Survey. II: Cohort effects. Journal of Affective Disorders, 30(1), 15–26. 8151045 10.1016/0165-0327(94)90147-3

[brb32384-bib-0058] Khalid, I. , Khalid, T. J. , Qabajah, M. R. , Barnard, A. G. , & Qushmaq, I. A. (2016). Healthcare workers emotions, perceived stressors and coping strategies during a MERS‐CoV outbreak. Clinical Medicine & Research, 14(1), 7–14. 10.3121/cmr.2016.1303 26847480 PMC4851451

[brb32384-bib-0059] Kim‐Godwin, Y. S. , Lee, M. H. , Logan, J. G. , & Liu, X. (2021). Factors influencing sleep quality among female staff nurses during the early COVID‐19 pandemic in the United States. International Journal of Environmental Research and Public Health, 18(9), 4827. 10.3390/ijerph18094827 33946606 PMC8124220

[brb32384-bib-0060] Kwan, M. Y. , Gordon, K. H. , Eddy, K. T. , Thomas, J. J. , Franko, D. L. , & Troop‐Gordon, W. (2014). Gender differences in coping responses and bulimic symptoms among undergraduate students. Eating Behaviors, 15(4), 632–637. 10.1016/j.eatbeh.2014.08.020 25248128

[brb32384-bib-0061] Labrague, L. J. (2021). Pandemic fatigue and clinical nurses’ mental health, sleep quality and job contentment during the covid‐19 pandemic: The mediating role of resilience. Journal of Nursing Management. 10.1111/jonm.13383 PMC8237073 34018270

[brb32384-bib-0062] Lancee, J. , Spoormaker, V. I. , & van den Bout, J. (2010). Cognitive‐behavioral self‐help treatment for nightmares: A randomized controlled trial. Psychotherapy and Psychosomatics, 79(6), 371–377. 10.1159/000320894 20829648

[brb32384-bib-0063] Lazarus, R. (2006). Stress and emotion: A new synthesis. Springer Publishing Company.

[brb32384-bib-0064] Lee, S. , Buxton, O. M. , Andel, R. , & Almeida, D. M. (2019). Bidirectional associations of sleep with cognitive interference in employees’ work days. Sleep Health, 5(3), 298–308. 10.1016/j.sleh.2019.01.007 30905693 PMC11487484

[brb32384-bib-0065] Lee, S. M. , Kang, W. S. , Cho, A.‐R. , Kim, T. , & Park, J. K. (2018). Psychological impact of the 2015 MERS outbreak on hospital workers and quarantined hemodialysis patients. Comprehensive Psychiatry, 87, 123–127. 10.1016/j.comppsych.2018.10.003 30343247 PMC7094631

[brb32384-bib-0066] Leung, G. M. , Lam, T.‐H. , Ho, L.‐M. , Ho, S.‐Y. , Chan, B. H. Y. , Wong, I. O. L. , & Hedley, A. J. (2003). The impact of community psychological responses on outbreak control for severe acute respiratory syndrome in Hong Kong. Journal of Epidemiology and Community Health, 57(11), 857–863. 10.1136/jech.57.11.857 14600110 PMC1732323

[brb32384-bib-0067] Li, G. , Kong, L. , Zhou, H. , Kang, X. , Fang, Y. , & Li, P. (2016). Relationship between prenatal maternal stress and sleep quality in Chinese pregnant women: The mediation effect of resilience. Sleep Medicine, 25, 8–12. 10.1016/j.sleep.2016.02.015 27823722

[brb32384-bib-0068] Li, Y. , Gu, S. , Wang, Z. , Li, H. , Xu, X. , Zhu, H. , Deng, S. , Ma, X. , Feng, G. , Wang, F. , & Huang, J. H. (2019). Relationship between stressful life events and sleep quality: Rumination as a mediator and resilience as a moderator. Frontiers in Psychiatry, 10, 348. 10.3389/fpsyt.2019.00348 31191370 PMC6545794

[brb32384-bib-0069] Li, Y. , Qin, Q. , Sun, Q. , Sanford, L. D. , Vgontzas, A. N. , & Tang, X. (2020). Insomnia and psychological reactions during the COVID‐19 outbreak in China. Journal of Clinical Sleep Medicine, 16(8), 1417–1418. 10.5664/jcsm.8524 32351206 PMC7446072

[brb32384-bib-0070] Liang, L. , Ren, H. , Cao, R. , Hu, Y. , Qin, Z. , Li, C. , & Mei, S. (2020). The effect of COVID‐19 on youth mental health. The Psychiatric Quarterly, 91(3), 841–852. 10.1007/s11126-020-09744-3 32319041 PMC7173777

[brb32384-bib-0071] Lin, L.‐Y. , Wang, J. , Ou‐Yang, X.‐Y. , Miao, Q. , Chen, R. , Liang, F.‐X. , Zhang, Y.‐P. , Tang, Q. , & Wang, T. (2021). The immediate impact of the 2019 novel coronavirus (COVID‐19) outbreak on subjective sleep status. Sleep Medicine, 77, 348–354. 10.1016/j.sleep.2020.05.018 32593614 PMC7831667

[brb32384-bib-0072] Liu, X. , Liu, C. , Tian, X. , Zou, G. , Li, G. , Kong, L. , & Li, P. (2016). Associations of perceived stress, resilience and social support with sleep disturbance among community‐dwelling adults. Stress and Health, 32(5), 578–586. 10.1002/smi.2664 26669814

[brb32384-bib-0073] Long, R. , Kennedy, M. , Malloy Spink, K. , & Lengua, L. J. (2021). Evaluation of the implementation of a well‐being promotion program for college students. Frontiers in Psychiatry, 12, 610931. 10.3389/fpsyt.2021.610931 33643091 PMC7907426

[brb32384-bib-0074] Majeno, A. , Tsai, K. M. , Huynh, V. W. , McCreath, H. , & Fuligni, A. J. (2018). Discrimination and sleep difficulties during adolescence: The mediating roles of loneliness and perceived stress. Journal of Youth and Adolescence, 47(1), 135–147. 10.1007/s10964-017-0755-8 29164378 PMC5750084

[brb32384-bib-0075] Mallampalli, M. P. , & Carter, C. L. (2014). Exploring sex and gender differences in sleep health: A society for women's health research report. Journal of Women's Health, 23(7), 553–562. 10.1089/jwh.2014.4816 PMC4089020 24956068

[brb32384-bib-0076] Matthews, K. A. , Hall, M. H. , Cousins, J. , & Lee, L. (2016). Getting a good night's sleep in adolescence: Do strategies for coping with stress matter? Behavioral Sleep Medicine, 14(4), 367–377. 10.1080/15402002.2015.1007994 26371884 PMC4792790

[brb32384-bib-0077] McCanlies, E. C. , Mnatsakanova, A. , Andrew, M. E. , Burchfiel, C. M. , & Violanti, J. M. (2014). Positive psychological factors are associated with lower PTSD symptoms among police officers: Post Hurricane Katrina. Stress and Health, 30(5), 405. 10.1002/smi.2615 25476965 PMC4676265

[brb32384-bib-0078] McEwen, B. S. (2000). The neurobiology of stress: From serendipity to clinical relevance. Brain Research, 886(1–2), 172–189. 10.1016/S0006-8993(00)02950-4 11119695

[brb32384-bib-0079] McKay, D. , Yang, H. , Elhai, J. , & Asmundson, G. J. G. (2020). Anxiety regarding contracting COVID‐19 related to interoceptive anxiety sensations: The moderating role of disgust propensity and sensitivity. Journal of Anxiety Disorders, 73, 102233. 10.1016/j.janxdis.2020.102233 32442880 PMC7194061

[brb32384-bib-0080] Meng, X. , & D'Arcy, C. (2015). Coping strategies and distress reduction in psychological well‐being? A structural equation modelling analysis using a national population sample. Epidemiology and Psychiatric Sciences, 25(4), 370–383. 10.1017/S2045796015000505 26077164 PMC7137609

[brb32384-bib-0081] *Mental Health—Household Pulse Survey—COVID‐19* . (2021). https://www.cdc.gov/nchs/covid19/pulse/mental‐health.htm

[brb32384-bib-0082] Milojevich, H. M. , & Lukowski, A. F. (2016). Sleep and mental health in undergraduate students with generally healthy sleep habits. Plos One, 11(6), e0156372. 10.1371/journal.pone.0156372 27280714 PMC4900547

[brb32384-bib-0083] Mohammadzadeh, F. , Noghabi, A. D. , Khosravan, S. , Bazeli, J. , Armanmehr, V. , & Paykani, T. (2020). Anxiety severity levels and coping strategies during the COVID‐19 pandemic among people aged 15 years and above in Gonabad, Iran. Archives of Iranian Medicine, 23(9), 633–638. 10.34172/aim.2020.76 32979912

[brb32384-bib-0084] Molock, S. D. , & Parchem, B. (2021). The impact of COVID‐19 on college students from communities of color. Journal of American College Health, 1–7. 10.1080/07448481.2020.1865380 33502970

[brb32384-bib-0085] Mong, J. A. , & Cusmano, D. M. (2016). Sex differences in sleep: Impact of biological sex and sex steroids. Philosophical Transactions of the Royal Society B: Biological Sciences, 371(1688). 10.1098/rstb.2015.0110 PMC4785896 26833831

[brb32384-bib-0086] Morin, C. M. , Rodrigue, S. , & Ivers, H. (2003). Role of stress, arousal, and coping skills in primary insomnia. Psychosomatic Medicine, 65(2), 259–267. 10.1097/01.psy.0000030391.09558.a3 12651993

[brb32384-bib-0087] Nestler, E. J. , Barrot, M. , DiLeone, R. J. , Eisch, A. J. , Gold, S. J. , & Monteggia, L. M. (2002). Neurobiology of depression. Neuron, 34(1), 13–25. 11931738 10.1016/s0896-6273(02)00653-0

[brb32384-bib-0088] Nicola, M. , Alsafi, Z. , Sohrabi, C. , Kerwan, A. , Al‐Jabir, A. , Iosifidis, C. , Agha, M. , & Agha, R. (2020). The socio‐economic implications of the coronavirus pandemic (COVID‐19): A review. International Journal of Surgery, 78, 185–193. 10.1016/j.ijsu.2020.04.018 32305533 PMC7162753

[brb32384-bib-0089] Ohayon, M. M. , Reynolds, C. F. , & Dauvilliers, Y. (2013). Excessive sleep duration and quality of life. Annals of Neurology, 73(6), 785–794. 10.1002/ana.23818 23846792 PMC4142503

[brb32384-bib-0090] Ortega, P. , Martínez, G. , & Diamond, L. (2020). Language and health equity during COVID‐19: Lessons and opportunities. Journal of Health Care for the Poor and Underserved, 31(4), 1530–1535. 10.1353/hpu.2020.0114 33416734 PMC9165570

[brb32384-bib-0091] Paiva, T. , Reis, C. , Feliciano, A. , Canas‐Simião, H. , Machado, M. A. , Gaspar, T. , Tomé, G. , Branquinho, C. , Silva, M. R. , Ramiro, L. , Gaspar, S. , Bentes, C. , Sampaio, F. , Pinho, L. , Pereira, C. , Carreiro, A. , Moreira, S. , Luzeiro, I. , Pimentel, J. , … Matos, M. G. (2021). Sleep and awakening quality during COVID‐19 confinement: Complexity and relevance for health and behavior. International Journal of Environmental Research and Public Health, 18(7), 3506. 10.3390/ijerph18073506 33800607 PMC8037491

[brb32384-bib-0092] Palagini, L. , Moretto, U. , Novi, M. , Masci, I. , Caruso, D. , Drake, C. L. , & Riemann, D. (2018). Lack of resilience is related to stress‐related sleep reactivity, hyperarousal, and emotion dysregulation in insomnia disorder. Journal of Clinical Sleep Medicine, 14(5), 759–766. 10.5664/jcsm.7100 29734989 PMC5940426

[brb32384-bib-0093] Peach, H. , Gaultney, J. F. , & Gray, D. D. (2016). Sleep hygiene and sleep quality as predictors of positive and negative dimensions of mental health in college students. Cogent Psychology, 3, 1168768. 10.1080/23311908.2016.1168768

[brb32384-bib-0094] Pepin, J. R. , Sayer, L. C. , & Casper, L. M. (2018). Marital status and mothers’ time use: Childcare, housework, leisure, and sleep. Demography, 55(1), 107–133. 10.1007/s13524-018-0647-x 29423629 PMC6560646

[brb32384-bib-0095] Peterson, L. M. , Miller, K. G. , Wong, P. M. , Anderson, B. A. , Kamarck, T. W. , Matthews, K. A. , Kirschbaum, C. , & Manuck, S. B. (2017). Sleep duration partially accounts for race differences in diurnal cortisol dynamics. Health Psychology, 36(5), 502–511. 10.1037/hea0000468 28425739 PMC5505864

[brb32384-bib-0096] Pfefferbaum, B. , & North, C. S. (2020). Mental health and the Covid‐19 pandemic. New England Journal of Medicine, 383, 510–512. 10.1056/NEJMp2008017 32283003

[brb32384-bib-0097] Pillai, V. , Roth, T. , Mullins, H. M. , & Drake, C. L. (2014). Moderators and mediators of the relationship between stress and insomnia: Stressor chronicity, cognitive intrusion, and coping. Sleep, 37(7), 1199–1208. 10.5665/sleep.3838 25061248 PMC4098805

[brb32384-bib-0098] Prolific website . https://www.prolific.co/

[brb32384-bib-0099] Qualtrics website . (2000) https://www.qualtrics.com/Accessed

[brb32384-bib-0100] Ramalingaswami, V. (2001). Psychosocial effects of the 1994 plague outbreak in Surat, India. Military Medicine, 166(2), 29–30. 10.1093/milmed/166.suppl_2.29 11778425

[brb32384-bib-0101] Ramsawh, H. J. , Bomyea, J. , Stein, M. B. , Cissell, S. H. , & Lang, A. J. (2016). Sleep quality improvement during cognitive behavioral therapy for anxiety disorders. Behavioral Sleep Medicine, 14(3), 267–278. 10.1080/15402002.2014.981819 26244485 PMC4744149

[brb32384-bib-0102] Rodriguez‐Seijas, C. , Fields, E. C. , Bottary, R. , Kark, S. M. , Goldstein, M. R. , Kensinger, E. A. , Payne, J. D. , & Cunningham, T. J. (2020). Comparing the impact of COVID‐19‐related social distancing on mood and psychiatric indicators in sexual and gender minority (SGM) and non‐SGM individuals. Frontiers in Psychiatry, 11, 590318. 10.3389/fpsyt.2020.590318 33414732 PMC7783401

[brb32384-bib-0103] Rosenberg, H. J. , Jankowski, M. K. , Fortuna, L. R. , Rosenberg, S. D. , & Mueser, K. T. (2011). A pilot study of a cognitive restructuring program for treating posttraumatic disorders in adolescents. Psychological Trauma: Theory, Research, Practice, and Policy, 3(1), 94–99. 10.1037/a0019889

[brb32384-bib-0104] Ross, R. J. , Ball, W. A. , Sullivan, K. A. , & Caroff, S. N. (1989). Sleep disturbance as the hallmark of posttraumatic stress disorder. The American Journal of Psychiatry, 146(6), 697–707. 10.1176/ajp.146.6.697 2658624

[brb32384-bib-0031] Rudenstine, S. , McNeal, K. , Schulder , T. , Ettman , C. K. , Hernandez , M. , Gvozdieva , K. , Galea , S. (2021). Depression and anxiety during the COVID‐19 pandemic in an urban, low‐income public university sample. Journal of Traumatic Stress, 34, 12–22. https://onlinelibrary.wiley.com/doi/10.1002/jts.22600 10.1002/jts.22600 PMC7675401 33045107

[brb32384-bib-0105] Salfi, F. , Lauriola, M. , Amicucci, G. , Corigliano, D. , Viselli, L. , Tempesta, D. , & Ferrara, M. (2020). Gender‐related time course of sleep disturbances and psychological symptoms during the COVID‐19 lockdown: A longitudinal study on the Italian population. Neurobiology of Stress, 13, 100259. 10.1016/j.ynstr.2020.100259 33102641 PMC7572275

[brb32384-bib-0106] Saraswathi, I. , Saikarthik, J. , Kumar, K. S. , Srinivasan, K. M. , Ardhanaari, M. , & Gunapriya, R. (2020). Impact of COVID‐19 outbreak on the mental health status of undergraduate medical students in a COVID‐19 treating medical college: A prospective longitudinal study. PeerJ, 8, e10164. 10.7717/peerj.10164 33088628 PMC7571415

[brb32384-bib-0107] Seelig, A. D. , Jacobson, I. G. , Donoho, C. J. , Trone, D. W. , Crum‐Cianflone, N. F. , & Balkin, T. J. (2016). Sleep and health resilience metrics in a large military cohort. Sleep, 39(5), 1111–1120. 10.5665/sleep.5766 26951391 PMC4835310

[brb32384-bib-0108] Seravalle, G. , Mancia, G. , & Grassi, G. (2018). Sympathetic nervous system, sleep, and hypertension. Current Hypertension Reports, 20(9), 74. 10.1007/s11906-018-0874-y 29980938

[brb32384-bib-0109] Shatkin, J. P. , Diamond, U. , Zhao, Y. , DiMeglio, J. , Chodaczek, M. , & Bruzzese, J.‐M. (2016). Effects of a risk and resilience course on stress, coping skills, and cognitive strategies in college students. Teaching of Psychology, 43(3), 204–210. 10.1177/0098628316649457

[brb32384-bib-0110] Sherwood, A. , Routledge, F. S. , Wohlgemuth, W. K. , Hinderliter, A. L. , Kuhn, C. M. , & Blumenthal, J. A. (2011). Blood pressure dipping: Ethnicity, sleep quality, and sympathetic nervous system activity. American Journal of Hypertension, 24(9), 982–988. 10.1038/ajh.2011.87 21633397 PMC3638212

[brb32384-bib-0111] Shrivastava, A. , De Sousa, A. , & Lodha, P. (2019). Resilience as a psychopathological construct for psychiatric disorders. In Y.‐K. Kim (Ed.), Frontiers in psychiatry (pp. 479–489). Springer. 10.1007/978-981-32-9721-0_23 31705509

[brb32384-bib-0112] Siddique, R. F. , Ahmed, O. , & Hossain, K. N. (2021). Relationship between the fear of COVID‐19 disease and sleep quality: The mediating role of stress. Heliyon, 7(5), e07033. 10.1016/j.heliyon.2021.e07033 34027200 PMC8123159

[brb32384-bib-0113] Slopen, N. , Lewis, T. T. , & Williams, D. R. (2016). Discrimination and sleep: A systematic review. Sleep Medicine, 18, 88–95. 10.1016/j.sleep.2015.01.012 25770043 PMC4699868

[brb32384-bib-0114] Smith, B. W. , Dalen, J. , Wiggins, K. , Tooley, E. , Christopher, P. , & Bernard, J. (2008). The brief resilience scale: Assessing the ability to bounce back. International Journal of Behavioral Medicine, 15(3), 194–200. 10.1080/10705500802222972 18696313

[brb32384-bib-0115] Steinhardt, M. , & Dolbier, C. (2008). Evaluation of a resilience intervention to enhance coping strategies and protective factors and decrease symptomatology. Journal of American College Health, 56(4), 445–453. 10.3200/JACH.56.44.445-454 18316290

[brb32384-bib-0116] Szcześniak, D. , Gładka, A. , Misiak, B. , Cyran, A. , & Rymaszewska, J. (2021). The SARS‐CoV‐2 and mental health: From biological mechanisms to social consequences. Progress in Neuro‐Psychopharmacology & Biological Psychiatry, 104, 110046. 10.1016/j.pnpbp.2020.110046 32730915 PMC7384993

[brb32384-bib-0117] Tada, A. (2017). The associations among psychological distress, coping style, and health habits in Japanese nursing students: A cross‐sectional study. International Journal of Environmental Research and Public Health, 14(11), 1434. 10.3390/ijerph14111434 PMC5708073 29165395

[brb32384-bib-0118] Tanaka, H. , & Shirakawa, S. (2004). Sleep health, lifestyle and mental health in the Japanese elderly—Ensuring sleep to promote a healthy brain and mind. Journal of Psychosomatic Research, 56(5), 465–477. 10.1016/j.jpsychores.2004.03.002 15172202

[brb32384-bib-0119] Tobin, D. L. , Holroyd, K. A. , Reynolds, R. V. , & Wigal, J. K. (1989). The hierarchical factor structure of the Coping Strategies Inventory. Cognitive Therapy and Research, 13(4), 343–361. 10.1007/BF01173478

[brb32384-bib-0120] Tousignant, O. H. , Taylor, N. D. , Suvak, M. K. , & Fireman, G. D. (2019). Effects of rumination and worry on sleep. Behavior Therapy, 50(3), 558–570. 10.1016/j.beth.2018.09.005 31030873

[brb32384-bib-0121] Trammell, P. , Janet P. , Joseph, P. , Nataria T. , & Harriger, P. , Jennifer A. (2021). Racial and ethnic minority disparities in COVID‐19 related health, health beliefs and behaviors, and well‐being among students. Journal of American College Health, 1–7. 10.1080/07448481.2021.1890606 33759734

[brb32384-bib-0122] Tsang, S. , Avery, A. R. , Seto, E. Y. W. , & Duncan, G. E. (2021). Is COVID‐19 keeping us up at night? Stress, anxiety, and sleep among adult twins. Frontiers in Neuroscience, 15, 665777. 10.3389/fnins.2021.665777 33981199 PMC8107288

[brb32384-bib-0123] Ulrich, A. K. , Full, K. M. , Cheng, B. , Gravagna, K. , Nederhoff, D. , & Basta, N. E. (2021). Stress, anxiety, and sleep among college and university students during the COVID‐19 pandemic. Journal of American College Health, 1–5. 10.1080/07448481.2021.1928143 PMC8742838 34242544

[brb32384-bib-0124] Veenema, A. H. , Meijer, O. C. , Kloet, E. R. D. , & Koolhaas, J. M. (2003). Genetic selection for coping style predicts stressor susceptibility. Journal of Neuroendocrinology, 15(3), 256–267. 10.1046/j.1365-2826.2003.00986.x 12588514

[brb32384-bib-0125] Walker, D. M. , Cunningham, A. M. , Gregory, J. K. , & Nestler, E. J. (2019). Long‐term behavioral effects of post‐weaning social isolation in males and females. Frontiers in Behavioral Neuroscience, 13, 66. 10.3389/fnbeh.2019.00066 31031604 PMC6470390

[brb32384-bib-0126] Wang, C. , Pan, R. , Wan, X. , Tan, Y. , Xu, L. , Ho, C. S. , & Ho, R. C. (2020). Immediate psychological responses and associated factors during the initial stage of the 2019 coronavirus disease (COVID‐19) epidemic among the general population in China. International Journal of Environmental Research and Public Health, 17(5), 1729. 10.3390/ijerph17051729 PMC7084952 32155789

[brb32384-bib-0127] Wang, J. , Zhang, X. , Simons, S. R. , Sun, J. , Shao, D. , & Cao, F. (2020). Exploring the bi‐directional relationship between sleep and resilience in adolescence. Sleep Medicine, 73, 63–69. 10.1016/j.sleep.2020.04.018 32791441

[brb32384-bib-0128] Wang, X. , Chen, H. , Liu, L. , Liu, Y. , Zhang, N. , Sun, Z. , Lou, Q. , Ge, W. , Hu, B. , & Li, M. (2020). Anxiety and sleep problems of college students during the outbreak of COVID‐19. Frontiers in Psychiatry, 11, 588693. 10.3389/fpsyt.2020.588693 33329134 PMC7719633

[brb32384-bib-0129] Williams, M. T. , Metzger, I. W. , Leins, C. , & DeLapp, C. (2018). Assessing racial trauma within a DSM–5 framework: The UConn Racial/Ethnic Stress & Trauma Survey. Practice Innovations, 3(4), 242–260. 10.1037/pri0000076

[brb32384-bib-0130] Wood, S. K. , Walker, H. E. , Valentino, R. J. , & Bhatnagar, S. (2010). Individual differences in reactivity to social stress predicts susceptibility and resilience to a depressive phenotype: Role of corticotropin‐releasing factor. Endocrinology, 151(4), 1795–1805. 10.1210/en.2009-1026 20160137 PMC2850230

[brb32384-bib-0131] Wright, K. P. , Linton, S. K. , Withrow, D. , Casiraghi, L. , Lanza, S. M. , Iglesia, H. de la , Vetter, C. , & Depner, C. M. (2020). Sleep in university students prior to and during COVID‐19 stay‐at‐home orders. Current Biology, 30(14), R797–R798. 10.1016/j.cub.2020.06.022 32693068 PMC7284257

[brb32384-bib-0132] Wu, G. , Feder, A. , Cohen, H. , Kim, J. J. , Calderon, S. , Charney, D. S. , & Mathé, A. A. (2013). Understanding resilience. Frontiers in Behavioral Neuroscience, 7, 10. 10.3389/fnbeh.2013.00010 23422934 PMC3573269

[brb32384-bib-0133] Xiong, J. , Lipsitz, O. , Nasri, F. , Lui, L. M. W. , Gill, H. , Phan, L. , Chen‐Li, D. , Iacobucci, M. , Ho, R. , Majeed, A. , & McIntyre, R. S. (2020). Impact of COVID‐19 pandemic on mental health in the general population: A systematic review. Journal of Affective Disorders, 277, 55–64. 10.1016/j.jad.2020.08.001 32799105 PMC7413844

[brb32384-bib-0134] Yu, H. Y. R. , Ho, S. C. , So, K. F. E. , & Lo, Y. L. (2005). The psychological burden experienced by Hong Kong midlife women during the SARS epidemic. Stress and Health, 21(3), 177–184. 10.1002/smi.1051

[brb32384-bib-0135] Zhang, W.‐J. , Yan, C. , Shum, D. , & Deng, C.‐P. (2020). Responses to academic stress mediate the association between sleep difficulties and depressive/anxiety symptoms in Chinese adolescents. Journal of Affective Disorders, 263, 89–98. 10.1016/j.jad.2019.11.157 31818801

[brb32384-bib-0136] Zoccola, P. M. , Dickerson, S. S. , & Lam, S. (2009). Rumination predicts longer sleep onset latency after an acute psychosocial stressor. Psychosomatic Medicine, 71(7), 771–775. 10.1097/PSY.0b013e3181ae58e8 19622710

