# Supplementary material for: Stress, coping, resilience, and sleep during the COVID‐19 pandemic: A representative survey study of US adults
Source: Brain Behav. 2021 Oct 17;11(11):e2384. doi: 10.1002/brb3.2384 (PMC8613418; doi:10.1002/brb3.2384)
Supplement: Supplementary file 1 — Supporting Information [file BRB3-11-e2384-s001.pdf]

Supplemental Table 1. Bivariate associations of pandemic-related stress of COVID-19 and coping with demographics and resilience.

Variable		Pandemic-Related Stress	Primary Control Engagement Coping	Secondary Control Engagement Coping	Disengagement Coping	Involuntary Engagement	Involuntary Disengagement
Age	*r*	**-.201**	-.025	-.005	**-.213**	**-.165**	**-.275**
	p	**<.0001**	.623	.923	**<.0001**	**.001**	**<.0001**
	n	**393**	393	393	**393**	**393**	**393**
Gender	*r*	-.098	**-.171**	-.031	**-.109**	-.093	**-.118**
	p	.052	**.001**	.542	**.030**	.064	**.019**
	n	393	**393**	393	**393**	393	**393**
Race/Ethnicity	*F*	.795	1.895	**3.008**	.805	.846	2.270
	p	.497	.130	**.030**	.492	.469	.080
	n	389	389	**389**	389	389	389
Subjective SES	*r*	**-.115**	.062	.074	**-.139**	**-.112**	**-.178**
	p	**.022**	.220	.141	**.006**	**.027**	**<.001**
	n	**393**	393	393	**393**	**393**	**393**
Education Level	*r*	-.017	**.125**	-.030	-.015	.017	-.009
	p	.731	**.013**	.548	.767	.734	.863
	n	393	**393**	393	393	393	393
Unemployed, Looking for Work	*r*	**.114**	-.010	-.024	.042	.020	.053
	p	**.024**	.839	.630	.411	.694	.291
	n	**393**	393	393	393	393	393
Objective Infection Rate	*r*	**.134**	.098	.062	**.102**	.065	**.116**
	p	**.008**	.054	.219	**.043**	.198	**.022**
	n	**392**	392	392	**392**	392	**392**
Objective Mortality Rate	*r*	.098	.072	.042	.072	.043	.079
	p	.057	.163	.418	.163	.402	.128
	n	375	375	375	375	375	375
Brief Resilience Scale	*r*	**-.333**	.060	**.371**	**-.253**	**-.445**	**-.394**
	p	**<.0001**	.239	**<.0001**	**<.0001**	**<.0001**	**<.0001**
	n	**393**	393	**393**	**393**	**393**	**393**

Notes: **Bold** signifies significance at $p < .05$. Gender (0 = woman; 1 = man); Employment status (0 = Not unemployed, looking for work; 1 = Unemployed, looking for work). Continuous variables are coded such that higher is “more/greater” of the variable. Race is a categorical variable and F-tests from ANOVAs are reported.
